# Supplementary material for: Modeling glioblastoma heterogeneity as a dynamic network of cell states
Source: Mol Syst Biol. 2021 Sep 16;17(9):e10105. doi: 10.15252/msb.202010105 (PMC8444284; doi:10.15252/msb.202010105)
Supplement: Supplementary file 6 — Source Data for Figure 5 [file MSB-17-e10105-s004.zip › Figure5A_sourcedata/GSEA_3017/hallmarks_stateC.GseaPreranked.1621934520804/HALLMARK_MTORC1_SIGNALING.html]

Details for gene set HALLMARK\_MTORC1\_SIGNALING[GSEA]

|  || Dataset | state13017 |
| Phenotype | NoPhenotypeAvailable |
| Upregulated in class | na\_neg |
| GeneSet | HALLMARK\_MTORC1\_SIGNALING |
| Enrichment Score (ES) | -0.33654243 |
| Normalized Enrichment Score (NES) | -1.5432941 |
| Nominal p-value | 0.080314964 |
| FDR q-value | 0.07098433 |
| FWER p-Value | 0.512 |
Table: GSEA Results Summary

  

Fig 1: Enrichment plot: HALLMARK\_MTORC1\_SIGNALING      
 Profile of the Running ES Score & Positions of GeneSet Members on the Rank Ordered List

  

| PROBE | GENE SYMBOL | GENE\_TITLE | RANK IN GENE LIST | RANK METRIC SCORE | RUNNING ES | CORE ENRICHMENT || 1 | RRM2 |  |  | 19 | 0.542 | 0.0270 | No |
| 2 | DHFR |  |  | 55 | 0.405 | 0.0108 | No |
| 3 | MCM4 |  |  | 189 | 0.259 | -0.1930 | No |
| 4 | STC1 |  |  | 210 | -0.255 | -0.1996 | No |
| 5 | ELOVL6 |  |  | 254 | -0.264 | -0.2454 | No |
| 6 | SQLE |  |  | 293 | -0.274 | -0.2814 | No |
| 7 | NAMPT |  |  | 306 | -0.280 | -0.2713 | No |
| 8 | DHCR7 |  |  | 322 | -0.285 | -0.2659 | No |
| 9 | PPP1R15A |  |  | 342 | -0.293 | -0.2666 | No |
| 10 | SLC2A1 |  |  | 383 | -0.311 | -0.3020 | Yes |
| 11 | IGFBP5 |  |  | 384 | -0.311 | -0.2674 | Yes |
| 12 | HMGCR |  |  | 418 | -0.334 | -0.2880 | Yes |
| 13 | LDLR |  |  | 419 | -0.334 | -0.2509 | Yes |
| 14 | SLC2A3 |  |  | 422 | -0.336 | -0.2171 | Yes |
| 15 | INSIG1 |  |  | 434 | -0.342 | -0.1984 | Yes |
| 16 | HSPA5 |  |  | 447 | -0.350 | -0.1805 | Yes |
| 17 | IDI1 |  |  | 451 | -0.353 | -0.1466 | Yes |
| 18 | CYP51A1 |  |  | 476 | -0.378 | -0.1465 | Yes |
| 19 | LDHA |  |  | 498 | -0.412 | -0.1374 | Yes |
| 20 | SCD |  |  | 504 | -0.431 | -0.0983 | Yes |
| 21 | BCAT1 |  |  | 511 | -0.438 | -0.0602 | Yes |
| 22 | SYTL2 |  |  | 513 | -0.439 | -0.0132 | Yes |
| 23 | CD9 |  |  | 515 | -0.443 | 0.0342 | Yes |
| 24 | HMGCS1 |  |  | 529 | -0.466 | 0.0632 | Yes |
| 25 | CTSC |  |  | 531 | -0.470 | 0.1136 | Yes |
Table: GSEA details [plain text format]

  

Fig 2: HALLMARK\_MTORC1\_SIGNALING: Random ES distribution      
 Gene set null distribution of ES for **HALLMARK\_MTORC1\_SIGNALING**
